# A New Trainee-Run Insomnia Treatment Service for Patients Under Community Mental Health Teams

**DOI:** 10.1192/bjo.2022.100

**Published:** 2022-06-20

**Authors:** Lauren Waterman, Michael Creed

**Affiliations:** 1 South London and Maudsley NHS Foundation Trust, London, United Kingdom; 2 King's College London, London, United Kingdom

## Abstract

**Aims:**

Chronic insomnia is a common mental disorder that severely impacts the quality of life of those affected, increases the risk of comorbid mental disorder and physical illness, and makes treatment of other mental disorders less effective. It is particularly common in patients under secondary care mental health services. Once chronic, insomnia rarely resolves spontaneously. Cognitive behavioural therapy for insomnia (CBT-I) is a highly effective treatment that is recommended by the UK's National Institute of Health and Care Excellence as the first-line treatment. Despite this, CBT-I is not universally available or accessible throughout the UK. Therefore, LW obtrained training at a sleep clinic and then initiated, ran and evaluated a new CBT-I service for existing community patients within her NHS trust.

**Methods:**

Patients received individual holistic assessments with a psychiatry trainee, a 5-session weekly virtual group intervention run by LW, and an individual 3-month follow-up. LW also trained other psychiatry trainees to run future groups, initially under supervision. Clinical rating scales were employed at initial assessment, following the final workshop and at follow-up.

**Results:**

Seven patients completed all sessions, with five completing a 3-month follow-up review. All had suffered with chronic insomnia for over 5 years. All had moderate-to-severe insomnia on the Insomnia Severity Index (ISI) at assessment (mean 21.3), which had improved by the end of treatment (mean 14.3). All patients seen at follow-up either no longer had insomnia or had sub-threshold insomnia on the ISI (mean 7.2). From assessment to post-treatment to follow-up, mean scores on the Dysfunctional Beliefs and Attitudes About Sleep Scale reduced from 6.6 to 3.7 to 2.5, and mean scores on the Clinical Outcomes in Routine Evaluation–Outcome Measure reduced from 2.18 to 2.01 to 1.27. Mean scores on the Work and Social Adjustment Scale reduced from 23.6 (severe impairment) at assessment to 7.0 (low impairment) at follow-up. Two patients stopped taking sleeping tablets during the treatment, and remained off them at follow-up.

**Conclusion:**

Group CBT-I can be a highly effective treatment for insomnia for patients under CMHT services. In this service run by a psychiatry trainee without formal sleep medicine or CBT qualifications, the efficacy of the intervention on insomnia symptoms, as well as on anxiety and depressive symptoms, was similar to the efficacies found in clinical trials and in specialist sleep clinics. CBT-I can be easily learnt by psychiatry trainees and likely other professionals with psychological expertise, which could increase the availability and accessibility of this effective treatment.

